# Cerebrospinal Fluid Classical Biomarker Levels in Mixed vs. Pure A^+^T^+^ (A^+^T_1_^+^) Alzheimer’s Disease

**DOI:** 10.3390/biomedicines12122904

**Published:** 2024-12-20

**Authors:** Ioanna Tsantzali, Athanasia Athanasaki, Fotini Boufidou, Vasilios C. Constantinides, Maria-Ioanna Stefanou, Christos Moschovos, Christina Zompola, Sotirios G. Paraskevas, Anastasios Bonakis, Sotirios Giannopoulos, Georgios Tsivgoulis, Elisabeth Kapaki, George P. Paraskevas

**Affiliations:** 1 2nd Department of Neurology, “Attikon” General University Hospital, School of Medicine, National and Kapodistrian University of Athens, 12462 Athens, Greece; docjo1989@gmail.com (I.T.); athanasia.athan@yahoo.gr (A.A.); marianna421@hotmail.co.uk (M.-I.S.); moship@windowslive.com (C.M.); chriszompola@yahoo.gr (C.Z.); sotirispar5@gmail.com (S.G.P.); bonakistasos@med.uoa.gr (A.B.); sgiannop@uoi.gr (S.G.); tsivgoulisgiorg@yahoo.gr (G.T.); 2 Neurochemistry and Βiological Markers Unit, 1st Department of Neurology, “Eginition” Hospital, School of Medicine, National and Kapodistrian University of Athens, 11528 Athens, Greece; fboufidou@med.uoa.gr (F.B.); vconstan@med.uoa.gr (V.C.C.); ekapaki@med.uoa.gr (E.K.); 3 Department of Neurology, University of Tennessee Health Science Center, Memphis, TN 38163, USA

**Keywords:** Alzheimer’s disease, beta amyloid, tau protein, phospho-tau, cerebrospinal fluid, biomarkers

## Abstract

**Background**: Alzheimer’s disease (AD) may present with pure (typical or atypical) and mixed phenotypes, sometimes causing difficulties in (differential) diagnosis. In order to achieve a diagnostic accuracy as high as possible, the diagnosis of AD during life depends on various biomarkers, including the cerebrospinal fluid (CSF) biomarkers. **Methods**: Classical CSF AD biomarkers were determined in a total of 61 patients, classified as both beta amyloid- and tau-positive A^+^T^+^ (or A^+^T_1_^+^ according to the recently revised Alzheimer Association criteria for diagnosis and staging of AD). Twenty one of these patients fulfilled the criteria for mixed AD (mixed with Lewy bodies, cerebrovascular disease, or normal pressure hydrocephalus), whilst 40 had pure AD. Results: Patients did not differ with respect to gender, education, disease duration, and cognitive status. After controlling for confounding factors, no difference was observed between mixed and pure AD groups in Aβ_42_ or Aβ_42_/Aβ_40_ levels. Although by definition, patients of both groups had abnormal (increased) levels of phospho-tau_181_, the mixed AD group presented with lower (less abnormal) levels of phospho-tau181 and total tau as compared to the pure group. Conclusions: In patients with AD of comparable cognitive status, mixed AD cases may present with lower levels of tau proteins and, if close to the cut-off values, diagnostic uncertainty may be increased.

## 1. Introduction

Alzheimer’s disease (AD) is the most common cause of cognitive decline and dementia [1], characterized by both extracellular amyloid deposition in the form of amyloid plaques and intracellular hyperphosphorylated tau in the form of neurofibrillary tangles [2]. AD usually presents as an amnestic disorder of the hippocampal type [1,3], whilst atypical phenotypes may occur in roughly 15% of all AD patients [3,4] and in up to 22–64% of early-onset (pre-senile) patients [5], including, language (mainly logopenic-type) presentations, posterior cortical atrophy, frontal presentations and corticobasal syndrome [6]. However, mixed cases are not uncommon, in which cerebrovascular disease [7], Lewy body pathology [8], or normal pressure hydrocephalus (NPH) [9] may occur concomitantly to AD pathology. A clinically based diagnosis is not always accompanied by a high diagnostic accuracy. Ιt has been estimated that when the clinical diagnosis favors a non-AD disorder, there is a 39% chance for autopsy-proven (co)occurrence of AD [10]. On the other hand, when the clinical diagnosis favors AD, there is a ~30% chance of identifying a non-AD disorder at autopsy [11]. Thus, mixed presentations may represent a diagnostic challenge in clinical practice. Nevertheless, accurate in vivo diagnosis is needed for prognosis estimation and correct therapeutic decisions, especially in the era of disease-modifying treatments [12].

Cerebrospinal fluid (CSF) biomarkers are considered as a significant tool, useful in the (differential) diagnosis of AD during life [13]. “Classical” CSF AD biomarkers include amyloid peptide with 42 amino acids (Aβ_42_) or, better, the Aβ_42_/Aβ_40_ ratio as a marker of amyloid pathology (correlates negatively with amyloid burden), tau protein phosphorylated at threonine 181 (τ_P-181_) as a marker of tau pathology (correlated positively with tangle burden) and total tau protein as a marker of neuronal/axonal degeneration [14]. For more than 10 years, they have been incorporated in diagnostic and research criteria or guidelines for AD and other neurodegenerative disorders [3,4,15,16]. In 2018, the National Institute of Ageing—Alzheimer’s Association (NIA–AA) introduced the AT(N) classification system for the diagnostic classification of AD based on biomarkers [17]. The letter A indicates markers of amyloid pathology, T indicates markers of tau pathology (tangle formation), and N indicates markers of neurodegeneration (neuronal/axonal loss). Each letter is followed by either + or −, representing the positive (abnormal) or negative (normal) result of testing, respectively. The profile of AD is either A^+^T^+^(N)^+^ or A^+^T^+^(N)^−^ regardless of the type and severity of clinical symptoms [17]. Recently, the above classification has been revised by the Alzheimer’s Association Workgroup [18] and, among other revisions, τ_P-181_ which becomes abnormal early in AD progression as a result of amyloid plaque formation, is now categorized as T_1_. Indeed, CSF biomarkers have been proven useful in the identification of AD in patients with atypical presentations or in mixed cases with at least one [19] or, sometimes, more co-pathologies [20], including cases mixed with cerebrovascular pathology [7,21], Lewy body pathology [22,23] and normal pressure hydrocephalus [9,24].

Many studies concerning CSF biomarkers in dementia with Lewy bodies (DLB), vascular cognitive impairment, and idiopathic NPH have been conducted and reviewed, mainly dealing with the discrimination from AD and controls [25,26,27,28,29,30]. However, within AD, studies comparing pure vs. mixed AD cases are scarce [9,19,26], not allowing definite conclusions.

The aim of the present study was to perform a pilot study comparing the levels of classical CSF biomarkers between patients with pure (amnestic or atypical) AD and patients with mixed AD presentations.

## 2. Materials and Methods

### 2.1. Study Design

Criterion for inclusion was the presence of A^+^T^+^ CSF biomarker profile, which is compatible with AD according to the 2018 NIA-AA criteria [17], regardless of the clinical presentation. No control group was used since the aim was a comparison within AD patients and not with controls.

Exclusion criteria were (a) Presence of major systemic disorders, (b) Presence of secondary causes of cognitive decline, including neurosyphilis or any central nervous system infection, brain tumor, subdural hematoma, recent (within the last 6 months) stroke, and paraneoplastic or autoimmune encephalopathies. Normal pressure hydrocephalus was allowed, as was evidence of concomitant cerebrovascular or concomitant Lewy body pathology, and such patients were classified as mixed AD. (c) Vitamin B12 deficiency or hypothyroidism should be absent or restored for at least 6 months. (d) Contraindication for lumbar puncture including low platelet count or anticoagulants. (e) Other CSF biomarker profiles (including the A^+^T^−^ profile) were excluded.

Since this was a pilot study, no power analysis was performed prior to the study, and data analysis was executed at the completion of 2 years of consecutive patient recruitment.

**Figure 1:**
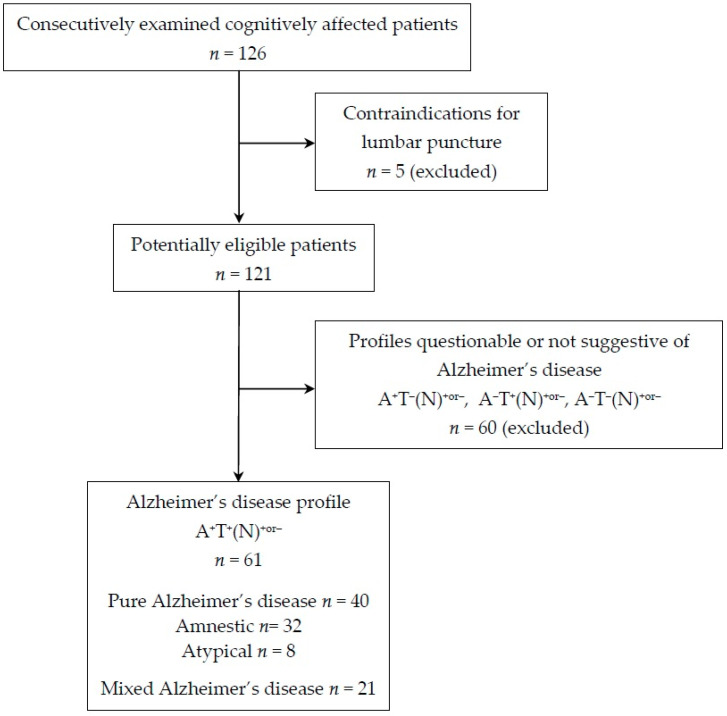
Flow chart of the participants included in the present study.

Finally, a total of 61 cognitively affected patients fulfilled the above criteria and were included in the study (Figure 1). They all showed the A^+^T^+^ CSF biomarker and were diagnosed with Alzheimer’s disease [17].

### 2.2. Patients

All patients were consecutively examined in the outpatient clinic and hospitalized in the 2nd Department of Neurology between March 2022 and March 2024. A written informed consent was provided by all subjects and/or their next of kin (in case of dementia). The study had the approval of the Ethics Committee and the Scientific Board of “Attikon” Hospital (project identification codes of approval: 157, 16 March 2021 and A13, 7 April 2021, respectively) and was conducted according to the ethical guidelines of the 1964 Declaration of Helsinki.

History, complete physical and neurological examination, structural neuroimaging, blood biochemical assays, and neuropsychological testing were all recorded. The Mini- Mental State Examination (MMSE) [31], which has been validated in Greece [32], was used as a crude estimate of the degree of cognitive decline. However, Addenbrooke’s Cognitive Examination-Revised version (ACE-R) [33], as adapted and validated in Greece [34], was preferred and used in the statistical analyses since it reflects better the global cognitive status, including not only learning and memory but also fluency, language and visuospatial abilities. Brain Magnetic Resonance Imaging or, when contraindicated, brain Computerized Tomography was used for the evaluation of atrophy and cerebrovascular burden. If indicated, a dopamine transporter scan by single-photon emission tomography (^123^I-FP-CIT SPECT) was performed.

### 2.3. Lumbar Puncture and CSF Biomarker Profiling

Lumbar puncture was performed using a standard, 21–22 G, Quincke-type needle at the L4–L5 interspace, and CSF was collected in 6 polypropylene tubes. The first three tubes were used for CSF cytology, biochemistry, syphilis testing, and oligoclonal band determination. The 4th and 5th tubes (5 mL each) were always used for biomarker determinations. CSF samples were collected and handled according to widely accepted recommendations on standardized operative procedures for CSF biomarkers [35] as described elsewhere [20]. All CSF samples had <500 red blood cells/μL. The two tubes intended for CSF biomarker analysis were immediately centrifuged (2000× *g*, 15 min), aliquoted in polypropylene tubes (1 mL each), and finally stored at −80 °C. Aliquots were thawed only once, just before analysis, which was performed within 3 months of storage.

Classical CSF biomarkers, namely Aβ_42_, Aβ_40_, phospho-tau-181 (τ_P-181_) and total tau (τ_T_) were measured at the Neurochemistry and Biological Markers Unit of the 1st Department of Neurology (“Eginition” Hospital), in a Euroimmun Analyzer I (Euroimmun, Lübeck, Germany), in duplicate, with double sandwich enzyme-linked immunosorbent assay (ELISA) by commercially available kits (EUROIMMUN Beta-Amyloid (1–42) ELISA, EUROIMMUN Beta-Amyloid (1–40) ELISA, EUROIMMUN pTau(181) ELISA and EUROIMMUN Total-Tau ELISA respectively), by the use of 4-parameter logistic curves and under stable temperature (21 ± 2 °C) with strict adherence to manufacturer’s instructions. Quality control samples (both in-house and provided by the manufacturer), including an in-house CSF pool, were used in every test run, resulting in inter- and intra-assay coefficients of variation < 7% and between-run precision > 90% for all biomarkers [36]. For external quality control, our laboratory participates in “The Alzheimer’s Association’s QC program for CSF and blood biomarkers” [37]. CSF biomarkers were considered normal according to cut-off values of the Neurochemistry and Biological Markers Unit, based on a large number of patients and normal controls (Aβ_42_ > 690 pg/mL, Aβ_42_/Aβ_40_ > 0.105, τ_P-181_ < 60 pg/mL, τ_T_ < 400 pg/mL) [20,36].

According to the CSF levels of biomarkers, the profile of each patient was determined according to the AT(N) classification system [15,16], and the AD profile was defined as A^+^T^+^. Amyloid positivity (A^+^) was defined as a decreased Aβ_42_/Aβ_40_ ratio since this ratio performs diagnostically better than Aβ_42_ alone [36,38]. However, in 6 patients (9.7%), Aβ_40_ was not available in order to calculate the above ratio, and decreased CSF levels of Aβ_42_ were used instead. Tau positivity (T^+^) was defined as increased CSF levels of τ_P-181_. Thus, according to the revised criteria of the Alzheimer’s Association Workgroup [18], the profile of patients included in the present study would be classified as A^+^T_1_^+^.

### 2.4. Patient Subgrouping

Following CSF biomarker profiling, the 61 patients with AD profile (A^+^T^+^) were subdivided into two groups based on their clinical presentation and according to the International Working Group IWG-2 criteria [4]. The pure AD group comprised 40 patients fulfilling the IWG-2 criteria for either amnestic (*n* = 32) or atypical AD (*n* = 8), including logopenic (*n* = 5) and posterior *(n* = 3) variants [4]. The mixed AD comprised 21 patients mixed with cerebrovascular disease (*n* = 11) and Lewy body disease (*n* = 4) [5]. In addition, all logopenic patients fulfilled the diagnostic criteria for an imaging-supported logopenic variant of primary progressive aphasia [39]. All patients with posterior variants fulfilled the Consensus Classification criteria for posterior cortical atrophy (pure) [40]. None of the pure AD patients showed possible non-AD signs, with pyramidal and pseudobulbar signs, stance and gait difficulties, urinary urgency or incontinence, parkinsonism (even mild), hallucinations, and fluctuations of cognitive status, all being completely absent. They also had no imaging signs indicative of significant cerebrovascular disease or NPH.

All mixed patients would otherwise fulfill the International Society for Vascular Behavioral and Cognitive Disorders (VASCOG) criteria for vascular cognitive disorder, with clinical and imaging features of significant subcortical small vessel disease and bilateral pyramidal and/or pseudobulbar signs [41] or the 4th consensus criteria for dementia with Lewy bodies with significant parkinsonism, visual hallucinations and fluctuations of cognitive status [42]. Furthermore, in the mixed AD group, 6 patients with probable idiopathic normal pressure hydrocephalus, according to the criteria of the Japanese Society of Normal Pressure Hydrocephalus [43] and concurrent A^+^T^+^ profile were added. They all had typical imaging signs of normal pressure hydrocephalus with Evans index > 0.31, focally enlarged sulci, and periventricular hyperintensities in addition to at least 2 features of cognitive decline, gait difficulty, and urinary urgency/incontinence.

## 3. Results

The 40 pure cases comprised 65.6% (95% confidence interval 46.9–89.3%) of all AD patients. The 21 mixed cases comprised 34.4% (95% confidence interval 21.3–52.6%) of all AD patients. Percentages of subgroups of the latter group were for mixed with cerebrovascular disease 18% (9–32.3%), for mixed with Lewy body disease 6.6% (1.8–16.8%) and mixed with normal pressure hydrocephalus 9.8% (3.6–21.4%). Results are summarized in Table 1.

### 3.1. Demographic and Clinical Data

Although males were underrepresented in the non-mixed as compared to the mixed AD group, the difference did not reach statistical significance. Age (at inclusion in the study) and age at disease onset were older in the mixed, as compared to the pure AD group (*t*-test, *p* = 0.0035 and *p* = 0.006, respectively).

Disease duration, education, MMSE, and ACE-R scores did not differ significantly between mixed and pure AD groups.

### 3.2. CSF Biomarkers

CSF Aβ_42_ levels did not differ between pure and mixed AD groups. A 2-way ANCOVA model (after logarithmic transformation) showed no significant effect by group, sex or sex by group, with disease duration, ACE-R score, and education having no significant effect on the model. However, age affected the model significantly, with younger patients showing lower levels of Aβ_42_ (beta 0.33, *p* = 0.019).

CSF Aβ_40_ levels were lower in mixed as compared to pure AD cases. A 2-way ANCOVA model (after logarithmic transformation) showed a significant effect by group (*p* = 0.03) but not by sex or sex by group, with disease duration and ACE-R score having no significance on the model. However, age and education affected the model significantly, with younger age and higher education being related to lower levels of Aβ_40_ (beta values 0.39 and −0.39 respectively, *p* = 0.006 and *p* = 0.004 respectively).

The Aβ_42_/Aβ_40_ ratio tended to be higher in mixed as compared to pure AD patients. (*t*-test, *p* = 0.06). After controlling for the various confounding factors, this marginal significance was lost, and a 2-way ANCOVA model showed no significant effect by group, sex, or sex by group, with age, disease duration, and ACE-R score having no significant effect on the model. However, education affected the model significantly, with lower education being related to a lower Aβ_42_/Aβ_40_ ratio (beta value 0.36, *p* = 0.009, respectively).

CSF levels of τ_P-181_ were lower in mixed as compared to pure AD cases. A 2-way ANCOVA model (after logarithmic transformation) showed a significant effect by group (*p* = 0.006), with no significant effect of sex, sex by group, age, disease duration, education, and ACE-R score.

CSF levels of τ_T_ were lower in mixed as compared to pure AD cases. A 2-way ANCOVA model (after logarithmic transformation) showed a significant effect by group (*p* = 0.019), with no significant effect of sex, sex by group, age, disease duration, and ACE-R score. Education had a significant effect on the model, with lower education being related to higher levels of τ_T_ (beta value −0.29, *p* = 0.03).

The τ_P-181_/Aβ_42_ ratio did not differ significantly between mixed and pure AD patients. A 2-way ANCOVA model showed no significant effect by group, sex, or sex by group, with disease duration and education and ACE-R having no significant on the model. Age affected the model significantly with older patients showing lower τ_P-181_/Aβ_42_ ratio (beta value −0.30, *p* = 0.026).

The τ_T_/Aβ_42_ ratio (after logarithmic transformation) did not differ significantly between mixed and pure AD groups, with no significant effect by any of the covariates.

The τ_P-181_/Aβ_40_ ratio did not differ significantly between mixed and pure AD groups. A 2-way ANCOVA model showed no significant effect by group, sex, or sex by group with no significant effect of disease duration, ACE-R, and education on the model. However, age affected the model significantly, with older age being related to a lower τ_P-181_/Aβ_40_ ratio (beta value −0.39, *p* = 0.006).

The τ_T_/Aβ_40_ ratio (after logarithmic transformation) did not differ significantly between mixed and pure AD groups, with no significant effect by any of the covariates.

The τ_P-181_/(Aβ_42_/Aβ_40_) ratio was significantly lower in mixed as compared to pure AD patients. A 2-way ANCOVA model (after logarithmic transformation) showed a significant effect by group (*p* = 0.008) but not sex, sex by group, age, disease duration, and ACE-R. However, education affected the model significantly, with lower education being related to a higher τ_P-181_/(Aβ_42_/Aβ_40_) ratio (beta value −0.36, *p* = 0.008).

The τ_T_/(Aβ_42_/Aβ_40_) ratio was significantly lower in mixed as compared to pure AD patients. A 2-way ANCOVA model (after logarithmic transformation) showed a significant effect by group (*p* = 0.015) but not sex, sex by group, age, disease duration, and ACE-R. However, education affected the model significantly, with lower education being related to a higher τ_T_/(Aβ_42_/Aβ_40_) ratio (beta value −0.42, *p* = 0.002).

**Figure 2:**
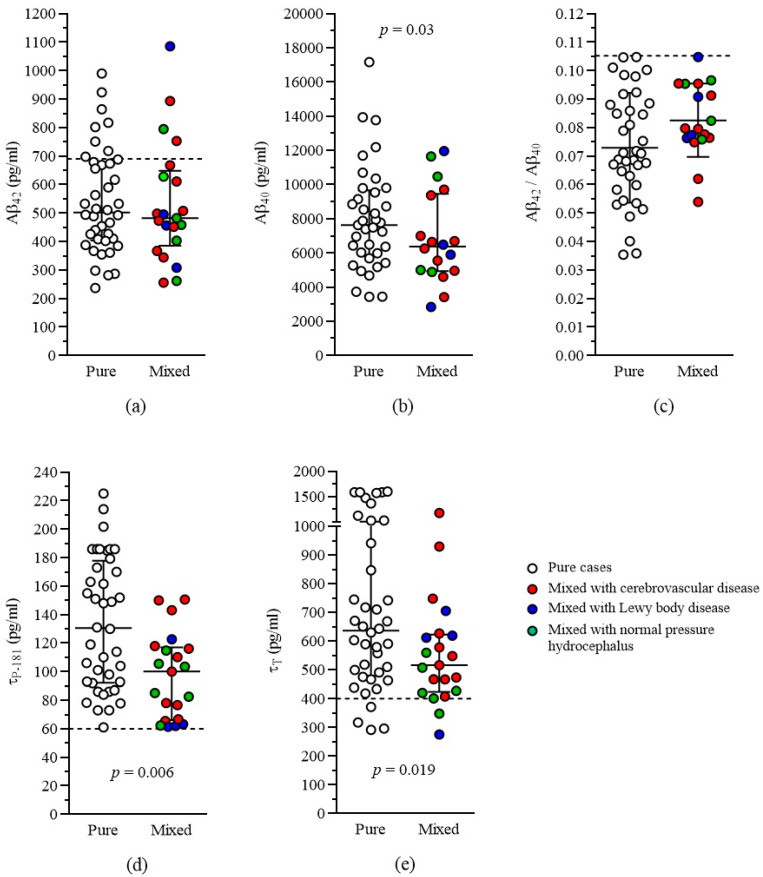
Scatterplot of CSF biomarker levels. Error bars for (**a**–**c**) indicate mean ± standard deviation. Error bars for (**d**,**e**) indicate median with interquartile range. Broken lines indicate cut-off values of our laboratory.

By visual inspection of Figure 2, within the mixed group, it seems that cases mixed with Lewy body disease may present with lower levels of τ_P-181_ and τ_T,_ whilst cases mixed with cerebrovascular disease may present with relatively higher levels. However, analysis of variance for comparison between the mixed subgroups revealed no significant results.

### 3.3. Correlations Between CSF Biomarkers

**Figure 3:**
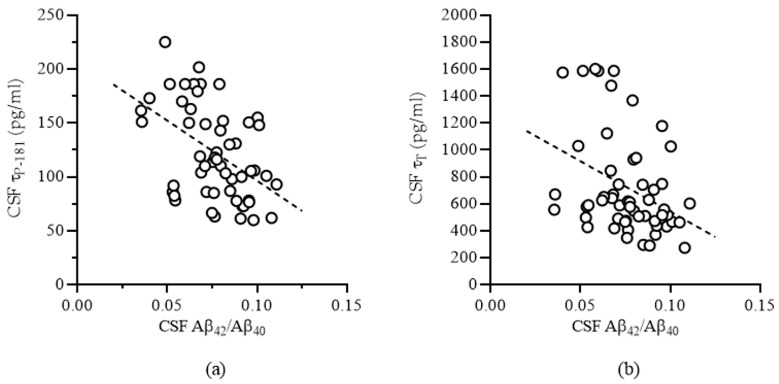
Negative correlation between the Aβ_42_/Aβ_40_ ratio and the CSF levels of τ_P-181_ (**a**) or τ_T_ (**b**).

Strong and statistically significant positive correlations were observed between Aβ_42_ and Aβ_40_, between τ_P-181_ and τ_T,_ and between τ_T_ and Aβ_40_. However, both τ_P-181_ and τ_T_ correlated negatively with the Aβ_42_/Aβ_40_ ratio (Table 2, Figure 3).

## 4. Discussion

In the present study, neither amyloid positivity alone nor the hybrid τ_P-181_/Aβ_42_ and/or τ_T_/Aβ_42_ ratios were used for AD diagnosis as suggested by the 2024 Alzheimer’s Association revised criteria [18]. It has been shown that the A^+^ profile is heterogeneous, comprising amnestic AD, mixed AD, and vascular cognitive impairment subgroups, with some of them showing diagnostic uncertainty [44], whilst some A^+^ individuals may not progress clinically [45]. In particular, the A^+^T^−^ profile may also be heterogeneous. Although many A^+^T^−^ patients may indeed suffer from AD [18], this profile provides no evidence of tangle pathology, which is necessary for the diagnosis of AD [2,17], whilst it can be observed in patients with non-AD pathologies, such as subcortical small vessel disease [21,46], including cerebral autosomal dominant arteriopathy with subcortical infarcts and leukoencephalopathy (CADASIL) [47], dementia with Lewy bodies (DLB) [22,30], limbic-predominant age-related TDP-43 encephalopathy [48], tauopathies with or without amyloid pathology [49,50], NPH [28,51] and in Creutzfeldt-Jakob disease [52]. It has been shown that a significant proportion of individuals with the A^+^T^−^ profile remain A^+^T^−^ for at least 5 years, and these individuals show lower *APOE* ε4 percentages and different neuropsychological and imaging characteristics as compared to A^+^T^+^ individuals [53]. On the other hand, the hybrid τ_P-181_/Aβ_42_ and τ_T_/Aβ_42_ ratios, although useful in the differential diagnosis of AD [54], including patients with conflicting results [55], may become abnormal due to very low levels of Aβ_42_ alone, without evidence of tau pathology. Thus, in order to obtain a high specificity of AD diagnosis and avoid contamination by strictly non-AD pathologies, we preferred the more conservative approach of the 2018 NIA-AA AT(N) classification system and the A^+^T^+^ profile [17], now called A^+^T_1_^+^ [18].

In the present study, we observed that in AD A^+^T^+^ (A^+^T_1_^+^) patients, those with mixed presentations had lower (less abnormal) levels of τ_P-181_ as compared to pure AD. Levels of τ_T_ were also lower in the mixed AD group, whilst no significant differences in either the Aβ_42_ or the Aβ_42_/Aβ_40_ ratio were observed. Indeed, in a recent study, it has been suggested that in A^+^ individuals, subgroups compatible with mixed AD or with a significant load of white matter hyperintensities show “uncertainty” with p-tau levels, which may deviate from the pattern typically expected for AD, becoming abnormal later in the course of the disease and sometimes being lower than expected or marginal [44].

In two previous studies of ours, we observed no significant difference between mixed vascular (vascular plus AD) and typical AD dementia [26] or between amnestic AD and mixed AD cases [19]. This discrepancy may be due to differences in methodology. In the first study [26], clinical criteria were used for diagnosis of vascular or mixed dementia. In the second [19], the criterion used for the diagnosis of AD was abnormal (low) Aβ_42_, abnormal (high) τ_P-181_, and abnormal (high) τ_T_ according to biomarker-based criteria introduced before the AT(N) system [56]. In the present study, an abnormal level of τ_T_ was not obligatory, according to the AT(N) system and, most importantly, the Aβ_42_/Aβ_40_ ratio was used as a marker of amyloid positivity instead of Aβ_42_ alone, which is considered as a better indicator of amyloid pathology [36,38]. Thus, more strict and robust criteria used may result in more accurate results. Noteworthy, in patients with vascular dementia based on clinical criteria, a previous study found that 53% of the patients had abnormal levels of Aβ_42_, but only 29% had abnormal levels of τ_P-181_ [57]. In an earlier study [58] (Herman et al., 2014), vascular patients with cognitive impairment were subdivided into two groups based on the Aβ_42_/Aβ_40_ ratio. In contrast to A^−^, patients classified as A^+^ had higher levels of τ_P-181_ as compared to controls whilst median τ_P-181_ concentration was increased to levels intermediate between AD and controls; however the difference did not reach statistical significance as compared to AD [58]. Thus, the design of the studies, with clinical criteria used for patient subgrouping and the use of Aβ_42_ alone as a marker of amyloid positivity, may lead to inconsistent results. Improvement of methodology over time with the use of robust biomarker-based criteria, including the Aβ_42_/Aβ_40_ ratio as a marker of amyloid positivity, may lead to more reliable results.

In mixed cases, lower levels of τ_P-181_ could be the result of the dual pathology. In our population, pure and mixed AD cases were at a comparable level of cognitive decline. In the presence of a co-pathology such as cerebrovascular or Lewy body disease, a lower load of AD lesions (and especially tangles) may be required in order to reach the same level of cognitive decline, resulting in less abnormal levels of τ_P-181_. In the presence of NPH, abnormal CSF dynamics may result not only in lower Aβ_42_ levels but additionally, may not allow a large increase of τ_P-181_ levels [29].

The decrease of Aβ_40_ observed in mixed AD is intriguing. Cerebral amyloid angiopathy (CAA) may result in lower levels of Aβ_40_ as compared to amnestic AD [59], and it is possible that some of the AD cases mixed with cerebrovascular disease could harbor CAA. However, this is only speculative since none of the patients fulfilled the Boston version 2.0 criteria for CAA [60].

The association of other pathologies, such as NPH, cerebrovascular disease, or Lewy pathology, with Alzheimer’s disease is being intensively investigated. It seems that in NPH, altered CSF dynamics and abnormal function of the glymphatic system may result in abnormal clearance and aggregation of proteins and peptides such as Aβ [61]. Cerebrovascular risk factors such as hypertension and diabetes are risk factors not only for vascular cognitive impairment but also for Alzheimer’s disease [62,63], whilst oxidative stress and neuroinflammation triggered by ischemia may lead to abnormal aggregation of proteins in brain tissue [64]. Finally, in Lewy body pathology, it seems that α-synuclein affects cognitive function at least partly by influencing tau pathology through interactions with molecules such as TNFR1 and ICAM-1 [65], and there may be a gradual development of AD pathology in patients initially harboring pure synucleinopathy [23]. On the other hand, many patients with AD are found at autopsy to have Lewy bodies as well [30], whilst AD may present with additional vascular disease through complex mechanisms, including CAA [59].

The present study has certain limitations. (1) There was no pathologic confirmation of the diagnosis in our groups and subgroups, and the diagnosis was based on the combination of clinical and CSF biomarker criteria. However, all patients are alive, and an autopsy cannot be available. (2) Only the classical CSF biomarkers were used for diagnosis of AD, whilst other neurodegenerative co-pathologies cannot be identified by inclusion. Additional fluid or imaging biomarkers of neurodegeneration, including α-synuclein [66,67,68] or TDP-43 [69,70], may prove helpful in the future. Furthermore, in case of marginally abnormal τ_P-181_ values, diagnostic doubts may occur and, in such cases, positron emission tomography for tau (tau-PET) may be of help [71]. Whether plasma p-tau217 would be an alternative solution remains to be established [72]. (3) The number of patients, especially in the subgroups of the mixed AD population, was low and an attempt for further comparisons between mixed subgroups resulted in non-significant results. This is not unusual in one-center investigations, which serve as pilot studies. Further studies with larger numbers of patients per (sub)group are necessary in order to demonstrate possible differences among subgroups (if any). However, at least for τ_P-181_, visual inspection of Figure 2d indicates that all 3 subgroups are distributed lower as compared to the pure AD group.

## Figures and Tables

**Table 1:** Demographic, clinical and CSF biomarker data of the studied groups.

	Pure AD	Mixed AD	*p* Value
*n* (m/f)	40 (12/28)	21 (11/10)	NS ^a^
Age (y)	68.6 ± 8.8	75.3 ± 6.9	0.0035 ^b^
Age at disease onset (y)	64.7 ± 9.7	71.5 ± 6.8	0.006 ^b^
Disease duration (y)	3.8 ± 3.5	3.8 ± 2.4	NS ^b^
Education (y)	10.6 ± 4.0	10.4 ± 4.0	NS ^b^
MMSE	21.1 ± 6.1	18.7 ± 4.5	NS ^b^
ACE-R	59.4 ± 18.2	52.8 ± 15.6	NS ^b^
Aβ_42_ (pg/mL)	502.4 (403.5–677.8) 541.5 ± 185.0	482.6 (385.2–648.0) 533.2 ± 211.5	NS ^c^
Aβ_40_ (pg/mL)	7633.0 (5834.0–9665.0) 7997.6 ± 3045.8	6373.0 (4939.0–9453.0) 6854.5 ± 2697.9	0.03 ^c^
Aβ_42_/Aβ_40_	0.0729 ± 0.0193	0.0826 ± 0.0129	NS ^d^
τ_P-181_ (pg/mL)	130.5 (92.2–177.7) 133.9 ± 45.9	100.1 (66.1–117.0) 97.1 ± 29.8	0.006 ^c^
τ_T_ (pgml)	636.4 (478.9–1005.0) 770.7 ± 407.0	516.0 (432.6–622.6) 562.4 ± 203.5	0.019 ^c^
τ_P-181_/Aβ_42_	0.2711 ± 0.1159	0.2040 ± 0.0851	NS ^d^
τ_T_/Aβ_42_	1.3240 (0.9478–1.8060) 1.5290 ± 0.8437	1.1200 (0.7882–1.4160) 1.1450 ± 0.4107	NS ^c^
τ_P-181_/Aβ_40_	0.0182 ± 0.0067	0.0164 ± 0.0064	NS ^d^
τ_T_/Aβ_40_	0.0917 (0.0628–0.1268) 0.1033 ± 0.0501	0.1037 (0.0609–0.1144) 0.0937 ± 0.0331	NS ^c^
τ_P-181_/(Aβ_42_/Aβ_40_) (pg/mL)	1624 (1169–2817) 2074 ± 1129	1188 (826–1551) 1250 ± 465	0.008 ^c^
τ_T_/(Aβ_42_/Aβ_40_) (pg/mL)	9809 (5691–17345) 12282 ± 8664	6870 (5366–8002) 7135 ± 2507	0.015 ^c^

Data are presented as mean ± standard deviation (for normally distributed data) or, in addition, as median values (25th–75th percentile) for data with deviations from normality. m: males, f: females, y: years, AD: Alzheimer’s disease, MMSE: Mini Mental State Examination, ACE-R Adenbrook’s Cognitive Examination Revised version. ^a^ Fisher’s exact test. ^b^ *t*-test. ^c^ 2-way ANCOVA with group and sex as co-factors and age, disease duration, education, and ACE-R score as covariates following the logarithmic transformation of original data. ^d^ 2-way ANCOVA with group and sex as co-factors and age, disease duration, education, and ACE-R score as co-variates.

**Table 2:** Correlation between CSF biomarkers studied.

	Aβ_42_	Aβ_40_	Aβ_42_/Aβ_40_	τ_P-181_	τ_T_
Aβ_42_	−	r = 0.692 *p* < 0.001	−	NS	NS
Aβ_40_	r = 0.692 *p* < 0.001	−	−	NS	*ρ* = 0.423 *p* = 0.01
Aβ_42_/Aβ_40_	−	−	−	*ρ* = −0.469 *p* = 0.0024	*ρ* = −0.375 *p* = 0.038
τ_P-181_	NS	NS	*ρ* = −0.469 *p* = 0.0024	−	*ρ* = 0.697 *p* < 0.001
τ_T_	NS	*ρ* = 0.423 *p* = 0.01	*ρ* = −0.375 *p* = 0.038	*ρ* = 0.697 *p* < 0.001	−

r: Pearson’s correlation coefficient. *ρ*: Spearman’s rank correlation coefficient. *p* values are Bonferroni-corrected for multiple correlations.

## Data Availability

The data presented in this study are available upon request from the corresponding author. The data are not publicly available due to privacy restrictions.
